# Distinctive Traits of Four Apulian Traditional Agri-Food Product (TAP) Cheeses Manufactured at the Same Dairy Plant

**DOI:** 10.3390/foods11030425

**Published:** 2022-02-01

**Authors:** Giuseppe Celano, Giuseppe Costantino, Maria Calasso, Cinzia Randazzo, Fabio Minervini

**Affiliations:** 1 Department of Soil, Plant and Food Sciences, University of Bari Aldo Moro, Via Amendola 165/a, 70126 Bari, Italy; maria.calasso@uniba.it (M.C.); fabio.minervini@uniba.it (F.M.); 2 Department of Veterinary Medicine-Food Safety Section, University of Bari Aldo Moro, Via Valenzano, 70010 Bari, Italy; giuseppe.costantino@uniba.it; 3 Department of Agricultural, Food and Environment, University of Catania, 95123 Catania, Italy; cinzia.randazzo@unict.it

**Keywords:** cheese microbiota, cheese quality, cheese safety, cheesemaking technology, metagenetic analysis, natural whey starter cultures, TAP cheeses, traditional agri-food products, volatile organic compounds

## Abstract

This study aimed to highlight the distinctive features of four Traditional Agri-food Products (TAP), namely, Caprino, Pecorino, Vaccino, and Cacioricotta cheeses produced at the same dairy plant to reveal any possible relationships between their microbiological and biochemical characteristics. Two distinct natural whey starter (NWS) cultures were used during Caprino and Vaccino cheesemaking, whereas no starter was used for the other cheeses. Cacioricotta retained the highest concentrations of salt and residual carbohydrates. Lactic acid bacteria dominated the microbiota of the cheeses. Furthermore, staphylococci represented an additional dominant microbial population in Cacioricotta. Although culture-dependent analysis showed that the use of NWS cultures only slightly affected the microbial community of cheeses, 16S metagenetic analysis showed that *Lactobacillus helveticus* dominated both the NWS cultures and the corresponding Caprino and Vaccino cheeses. This analysis indicated that *Staphylococcus equorum* and *Streptococcus thermophilus* dominated Cacioricotta and Pecorino cheeses, respectively. The highest peptidase activities were found in either Caprino or Vaccino. Enzymes involved in the catabolism of free amino acids and esterase showed the highest activity in Pecorino cheese. Each cheese showed a distinct profile of volatile organic compounds, with Pecorino being the richest cheese in carboxylic acids, ketones, and esters, related to lipolysis. The results of this study contribute to valorizing and safeguarding these TAP cheeses, sustaining local farming.

## 1. Introduction

Nowadays, consumers living in Western countries show a dichotomic aptitude towards food tradition and innovation: on the one hand, they are genuinely interested in food innovation, and on the other hand, they are attracted by traditional food. The mistrust about food manufactured at the industrial level using cutting-edge technologies could be one of the reasons for the renewed interest in traditional food. It is undeniable that traditional food, along with typical food, besides being referenced starting blocks for food innovation, represent an invaluable legacy to be protected [1]. European Policy on agri-food quality includes the protection of agricultural and food products through appropriate designations, namely, Protected Designation of Origin (PDO), Protected Geographical Indication (PGI), and Traditional Specialty Guaranteed (TSG). These labels are explicitly meant to provide consumers with detailed information about a given product, thus protecting them from the risk of imitation [2].

Besides the above-mentioned labels, the Italian Ministry of Agriculture and Forest Politics (MIPAAF) defined the Traditional Agri-food Products (TAP or, elsewhere, TFPs; in Italian language, *Prodotti Agroalimentari Tradizionali*, *PAT*), as those agriculture or food products “obtained with processing methods, storage and maturation over time consolidated, homogeneous throughout the territory concerned, according to traditional rules, for a period not less than twenty-five years” [3]. Different to PDO/PGI/TSG products, TAP face some limitations: (i) small amounts of production; (ii) production is referred to limited, and sometimes fringe, areas; (iii) production protocols may be not in conformity with hygiene regulations and/or show slight variations within the same geographical area [4]. Within the last updated list of TAP, around 500 are represented by cheeses [5], perhaps the most heterogenous group of fermented food. The distinctive traits of a given cheese result from several factors, such as type of milk, production protocol, and microbiota [6]. Among the 17 TAP cheeses referred to the Apulia region (Southern Italy), we focused on Caprino, Pecorino, Vaccino, and Cacioricotta, which have been on the list since 2000 [7]. Caprino, manufactured from goats’ milk, is a long ripened hard cheese. Pecorino is the oldest Apulian cheese, strictly related to the seasonal transhumance from the Abruzzo mountains to the Apulian plain, named *Tavoliere delle Puglie*, extending from the foot of the Gargano promontory and the Dauno Apennine to the Adriatic Sea. Pecorino cheese is manufactured from ewes’ milk and has a semi-hard or hard texture, depending on the length of the ripening period. Vaccino cheese, manufactured from cows’ milk, is characterized by a semi-hard or hard texture. Cacioricotta cheese is manufactured from a blend of milk (often goats’, cows’ and/or ewes’ milk), frequently sold after short (two months) ripening. It is related to seasonal transhumance [8].

The European Commission, in view of a tentative labeling scheme for local farming, suggested that “a new label could add value to products generated from local agriculture if it went beyond direct sales and if Member States were to ensure that it is integrated with or linked to other measures” [9]. The adoption of the TAP label for traditional food products, such as cheese, could represent a concrete acceptance of this suggestion. However, first, it seems necessary to increase knowledge about TAP [4]. A very limited number of studies have focused on TAP cheeses [10,11]. Although those studies depicted an overall picture about the quality of some TAP cheeses, which were either provided by temporary aggregations of dairy farms or purchased from the retail market of dairies, they did not consider the detailed production protocols or the many biochemical and microbiological traits of each cheese.

This study aimed to highlight the distinctive traits of four different TAP cheeses (Caprino, Pecorino, Vaccino, Cacioricotta) manufactured at the same semi-industrial plant. For this purpose, the cheeses were analyzed at the end of ripening to assess the microbiota, enzymatic activities, degree of proteolysis, and concentrations of the main compounds involved in their sensory traits. We chose a cross-sectional experimental plan, rather than a longitudinal one (i.e., sampling cheeses throughout ripening time), because for ripened cheeses the highest degree of distinctiveness is especially found at the end of ripening. In addition, we chose sampling the four cheeses from the same dairy plant, rather than from different plants, aiming to minimize the influence of house microbiota on cheeses’ distinctive traits.

## 2. Materials and Methods

### 2.1. Cheese and Natural Whey Starter Sampling

**Figure 1:**
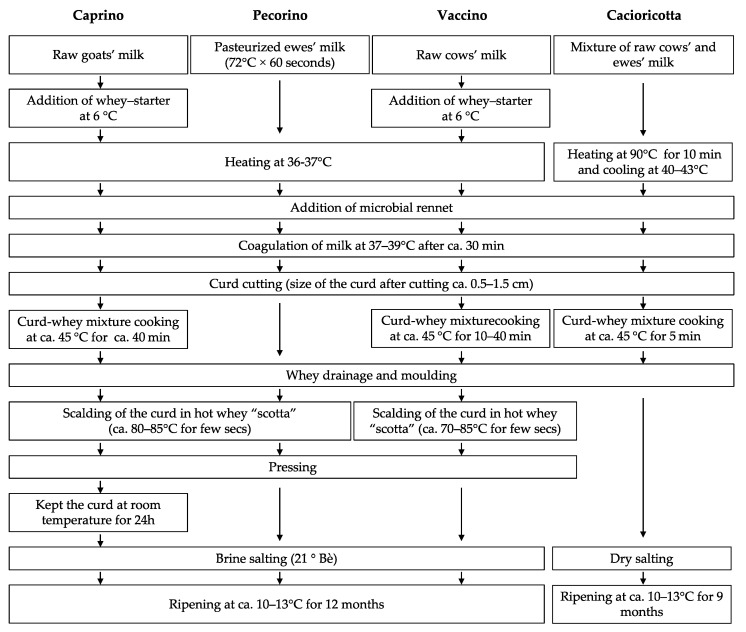
Protocols for the manufacture of Caprino, Pecorino, Vaccino, and Cacioricotta TAP cheeses.

Caprino (goats’ milk cheese), Pecorino (ewes’ milk cheese), Vaccino (cows’ milk cheese), and Cacioricotta (50% cows’ and ewes’ milk cheese) TAP cheeses were manufactured at a semi-industrial dairy plant located at Putignano, Bari, Southern Italy, according to traditional protocols (Figure 1), which were characteristic for each cheese, but shared the following aspects: (i) coagulation parameters were identical (including the microbial rennet used) for all the cheeses, except for a slightly higher renneting temperature adopted for curding Cacioricotta; (ii) except for Cacioricotta cheese, curd was scalded in the “scotta”, the hot, whey-based by-product of *ricotta* manufacturing; (iii) cheeses were salted in brine, except for Cacioricotta (dry salting); (iv) they were ripened at the same temperature.

Three batches were sampled for each cheese. Besides cheeses, natural whey starter (NWS) cultures used during production of Caprino (CNWS) and Vaccino (VNWS) cheeses were sampled.

Each sample of cheese and the NWS cultures was divided into two aliquots, one of which was stored at 4 °C and subjected (within 18 h from collection) to microbiological and biochemical analyses (primary and secondary proteolysis, residual enzymatic activities, volatile organic compounds). The second aliquot was stored at −80 °C and destined for 16S metagenetic analysis and profiling of volatile organic compounds (VOC).

### 2.2. Compositional Analysis

The moisture and residual solids content were evaluated using Moisture Analyzer MA35 (Sartorius Stedim Biotech GmbH, Göttingen, Germany). Water activity (A_w_) was determined through the Aqua Lab Decagon Dewires system (Pullman, WA, USA). The pH value was measured with a pH-meter equipped with a Foodtrode (Hamilton, Bonaduz, Switzerland) electrode. Cheeses were also analyzed for total carbohydrates through HPLC [12], proteins through Kjeldahl method [13], fat content [14], and salt [15]. The residual concentration of lactose and galactose was estimated through the Megazyme Lactose and D-Galactose assay kit K-LACGAR (Megazyme Int. Ireland Ltd., Bray, Ireland), following the producer’s instructions.

Concentration of lactic acid was estimated analyzing acid-soluble extract of cheese through HPLC Äkta Purifier System (GE Healthcare Biosciences, Uppsala, Sweden) equipped with a cation exchange column (Aminex HPX-87H), a Cation H+ Microguard (Bio-Rad-Laboratories, Hercules, CA, USA), and a UV detector (UV100) set at a wavelength of 210 nm. Acid-soluble extracts of cheese were obtained upon homogenization (through Stomacher, for 10 min) of five grams of cheese and 25 mL of H_2_SO_4_ 0.01 N. After centrifugation (2500× *g* for 5 min) of the homogenate, the supernatant, representing the cheese extract, was filtered using 0.20 µm pore size membrane filter (Sartorius AG, Gottingen, Germany) and injected in the column. Lactic acid was eluted isocratically with H_2_SO_4_ 0.01 N at a flow rate of 0.6 mL min^−1^, keeping the column at 60 °C [16].

### 2.3. Cultivable Microbiota

Ten grams of cheese were homogenized with 90 mL of sterile saline (NaCl, 9 g L^−1^) solution using a 400P Bag Mixer (180 s of treatment), while further serial dilutions were continued in Ringer quarter-strength solution and plated on different culture media purchased from Oxoid (Basingstoke, UK). Cell density of total mesophilic aerobic microorganisms was determined using Plate Count agar after incubation at 30 °C. Presumptive mesophilic lactobacilli and cocci were enumerated using de Man, Rogosa, and Sharpe (MRS) and lactose M17 agar plates, respectively, supplemented with cycloheximide (0.1% wt/vol), after incubation at 30 °C. Presumptive thermophilic lactobacilli and streptococci were enumerated on MRS and lactose M17 agar media, respectively, supplemented with cycloheximide (0.1% wt/vol) after incubation at 45 °C. Presumptive enterococci were enumerated on Slanetz and Bartley agar, after inoculating by spreading technique, and incubation of plates at 37 °C. Plates of Baird Parker agar, supplemented with egg yolk tellurite (5% vol/vol), inoculated by spreading and incubated at 37 °C, were used to enumerate presumptive staphylococci. Presumptive *Enterobacteriaceae* were counted on Violet Red Bile Glucose Agar (VRBGA) plates incubated at 37 °C. Plates of *Pseudomonas* agar, supplemented with cetrimide (10 mg L^−1^), Fucidin (10 mg L^−1^), and cephalosporin (50 mg L^−1^), were spread inoculated and used to enumerate presumptive *Pseudomonas* spp. after incubation at 30 °C. Yeasts were enumerated on Sabouraud Dextrose Agar plates supplemented with chloramphenicol (0.1% wt/vol) after incubation at 25 °C. The CNWS and VNWS cultures were diluted and plated on MRS and lactose M17 plates supplemented with cycloheximide (0.1% wt/vol) and were incubated as above for estimating cell densities of mesophilic and thermophilic lactobacilli and coccus-shaped lactic acid bacteria (LAB), respectively. All plates were incubated for 48 h, except for VRBGA and *Pseudomonas* agar, which were incubated for 24 h [17].

### 2.4. 16S Metagenetic Analysis

Total DNA from three batches of each cheese and NWS was extracted using the FastDNA Spin Kit (MP Biomedicals, Solon, OH, USA) according to the manufacturer’s instructions. All DNA samples were quantified by Nanodrop ND-1000 spectrophotometer (Thermo Fisher Scientific, Inc., Waltham, MA, USA). For each cheese and NWS, DNA was pooled [18] and used as a template for 16S metagenetic analysis, which was carried out at the Research and Testing Laboratory (RTL, Lubbock, TX, USA) by using the Illumina MiSeq platform. A fragment of the 16S rRNA gene for analysis of diversity inside the phylum of *Firmicutes* was amplified using the primers Firm350F/Firm814R [19]. Sequence data for each sample were processed using RTL’s in-house pipeline (https://rtlgenomics.com/documents (last accessed on 3 February 2020)). Briefly, the sequenced reads were (i) merged by PEAR Illumina paired-end merger [20]; (ii) trimmed using an internally developed quality trimming algorithm; (iii) grouped by using the USEARCH [21] algorithm into clusters (4% dissimilarity among sequences of the same cluster); (iv) the clusters were classified to Operational Taxonomic Unit (OTU) by using the UPARSE-OTU [22]; and finally checked using the UCHIME software [23]. The relative abundance of each bacterial OTU was analyzed individually within each sample [24].

### 2.5. Assessment of Enzymatic Activities

Water-soluble cheese extracts were prepared according to the method of Kuchroo and Fox [25]. The extracts were dialyzed (Dialysis Tubing, cut-off 12,000 Da, Sigma Company, Milan, Italy) for 24 h at 4 °C against 0.05 M phosphate buffer, pH 7.0, to eliminate interference from salt and peptides. To avoid interference due to cellular activity, the dialyzed extracts were subjected to sterile filtration (0.22 μm pore size, Syrfil Filter, Nucleopore, Costar Corporation, Cambridge, MA, USA) [26]. Aminopeptidase (EC 3.4.11.11) and proline iminopeptidase (EC 3.4.11.9) assays were carried out as described by Gobbetti et al. [26], using leu-*p*-nitroanilide and pro-*p*-nitroanilide as substrates. Endopeptidase type O (EC 3.4.23) was measured using Z-Gly-Pro-NH-trifluoromethyl coumarin as a substrate [27]. One unit of enzymatic activity was defined as the amount of enzyme that produced an increase in absorbance at 410 nm of 1 (aminopeptidase) and 0.1 (proline iminopeptidase and endopeptidase) AU min^−1^ at 37 °C and pH 7.0. Glutamate dehydrogenase (EC 1.4.1.2) activity was assayed by measuring the glutamate-dependent reduction in NADP^+^ or NAD^+^ at 492 nm [28]. One arbitrary unit of enzymatic activity was defined as the amount of enzyme that gave an increase in absorbance at 492 nm of 0.1 AU min^−1^ at 37 °C and pH 7.0. Cystathionine lyase (EC 4.4.1.1) activity was determined by measuring the amount of ketoacids, ammonia, and free thiols released from cystathionine [29]. One arbitrary unit of enzymatic activity was defined as the amount of enzyme that caused an increase (at 412 nm) of 1 AU min^−1^ at 37 °C and pH 7.0. Esterase (acetyl ester hydrolase, EC 3.1.1.6) activity was determined as described by Gobbetti et al. [30] using *β*-naphthyl butyrate as substrate and expressed as 1 μmol of *β*-naphthol released per min at 37 °C and pH 7.0.

### 2.6. Assessment of Proteolysis

The pH 4.6-soluble and pH 4.6-insoluble nitrogen fractions of cheeses were obtained according to the method described by Kuchroo and Fox [25]. The insoluble fraction was analyzed by denaturing urea polyacrylamide gel electrophoresis (urea-PAGE), using sodium caseinates from goats’, ewes’, and cows’ milk as standard references. The gels were stained using Coomassie Brilliant Blue G250, and destained according to Blakesley and Boezi [31]. Peptides contained in the pH 4.6-soluble nitrogen fraction were analyzed through an FPLC system (GE Healthcare Biosciences, Uppsala, Sweden) equipped with a Resource RPC reverse phase column and a UV detector operating at 214 nm [32]. The analysis was performed with a flow rate of 1 mL min^−1^ in gradient elution; the mobile phase consisted of water, acetonitrile, and trifluoroacetic acid (0.05%, *v/v*). The acetonitrile percentage was constantly increased from 5 to 46% between 16 min and 62 min from injection, to reach a final concentration of 100% between 62 and 72 min. The number and areas of peaks were recorded. Concentration of total free amino acids (FAA) in the pH 4.6-soluble fraction was determined through the cadmium-ninhydrin method [33] and expressed as mg g^−1^.

### 2.7. VOC Analysis

Four grams of grated cheese were added with 10 µL of internal standard solution (2-octanol, at 10 ppm), placed into 20 mL glass vials, and sealed with polytetrafluoroethylene-coated silicone rubber septa (20 mm diameter) (Supelco, Bellefonte, PA, USA). To obtain the best extraction efficiency, the micro-extraction procedure was performed as described in Salum et al. [34], with slight modifications. After sample equilibration (10 min at 54.75 °C), a conditioned 50/30 μm DVB/CAR/PDMS fiber (Supelco, Bellefonte, PA, USA) was exposed for 60 min. The temperature was kept constant during analysis, and the vials were maintained on a heater plate (CTC Analytics, Zwingen, Switzerland) of a CombiPAL system injector autosampler (CTC Analytics). The extracted VOC were desorbed in splitless mode (3 min at 220 °C) and analyzed through a Clarus 680 (Perkin Elmer) gas-chromatography (GC) system equipped with a capillary Rtx-WAX column (30 m × 0.25 mm i.d., 0.25 μm film thickness) (Restek, Bellfonte, PA, USA). The column temperature was set initially at 35 °C for 8 min, then increased to 60 °C at 4 °C min^−1^, to 160 °C at 6 °C min^−1^, and finally to 200 °C at 20 °C min^−1^ and held for 15 min. Helium was used as the carrier gas at flow rate of 1 mL min^−1^. A single quadrupole mass spectrometer (MS) Clarus SQ 8C (Perkin Elmer) was coupled to the GC system. The source and transfer line temperatures were kept at 250 and 230 °C, respectively. Electron ionization masses were recorded at 70 eV in the mass-to-charge ratio (*m/z*) interval 34–350 [35]. Each chromatogram was analyzed for peak identification using the NIST (National Institute of Standard and Technology) 2008 library. A peak area threshold of 1,000,000 and at least 85% probability of match were used for dentification, followed by visual inspection of the fragment patterns when required. The concentrations of VOC (calculated on internal standard base) were expressed as mg kg^−1^.

### 2.8. Statistical Analysis

Data collected from the analyses, performed at least in duplicate on three batches of cheese sampled at the same dairy plant, were subjected to one-way analysis of variance (ANOVA), and pair comparison of treatment means was achieved by Tukey’s procedure at *p* < 0.05, using the statistical software Statistica v. 7.0 for Windows. Principal component analysis (PCA) was also performed using Umetrics Simca 14.1.

## 3. Results

### 3.1. Compositional Analysis of TAP Cheeses

The highest and lowest moisture percentages were found in Cacioricotta (ca. 38%) and Pecorino (ca. 31%) cheeses, respectively (Table 1).

Fat content ranged from 27% (Cacioricotta) to ca. 35% (Pecorino); the same trend was found for protein content, varying between ca. 22% (Cacioricotta) to ca. 27% (Pecorino). Cacioricotta cheese was characterized by the highest (*p* < 0.05) amount of NaCl, whereas the other cheeses contained ca. 0.6 to ca. 0.9% of NaCl. Values of A_w_ ranged from ca. 0.86 (Vaccino) to ca. 0.90 (Cacioricotta). The highest (*p* < 0.05) pH value was found for Cacioricotta cheese (ca. 5.9), whereas no significant (*p* > 0.05) differences were found for the other cheeses (Table 1).

No residual lactose was detected in all the cheeses, except for Cacioricotta cheese (ca. 860 mg kg^−1^). The latter was also distinguished by the highest (*p* < 0.05) concentration of residual galactose, which was ca. 210 mg kg^−1^ vs. 20–50 mg kg^−1^ detected in the other cheeses. On the opposite, Cacioricotta cheese was characterized by the lowest (*p* < 0.05) concentration (ca. 2 mg kg^−1^) of lactic acid. The other cheeses contained 16–18 mg kg^−1^ of lactic acid, with no significant (*p* > 0.05) differences.

### 3.2. Cultivable Microbiota of TAP Cheeses

**Figure 2:**
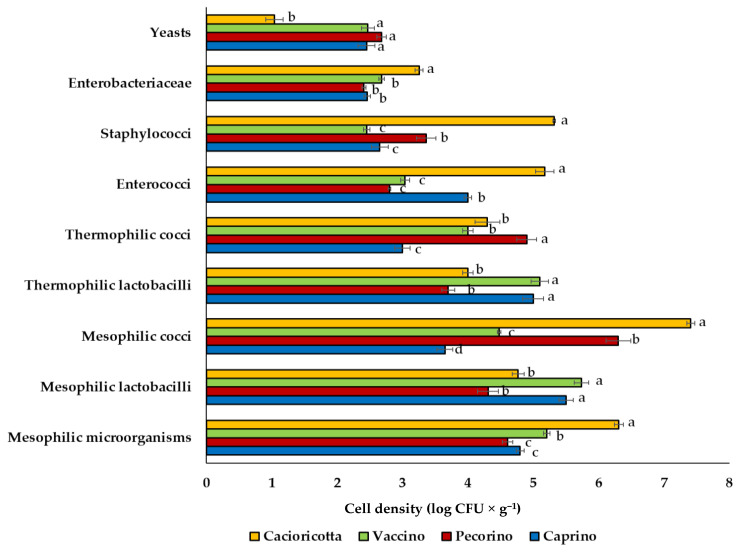
Cell densities (log CFU g^−1^) of different microbial groups found in Caprino, Pecorino, Vaccino, and Cacioricotta TAP cheeses. Bar-plots marked with different letters (a, b, c, d), within each microbial group, are significantly different (*p* < 0.05).

The cell density of total mesophilic microorganisms ranged from 4.6 (Pecorino) to 6.3 (Cacioricotta cheese) log CFU g^−1^ (Figure 2).

Presumptive mesophilic lactobacilli varied between 4.3 (Pecorino) and 5.7 (Vaccino cheese) log CFU g^−1^. The highest (*p* < 0.05) number of mesophilic coccus-shaped LAB was found in Cacioricotta cheese (ca. 7.4 log CFU g^−1^), whereas the lowest (*p* < 0.05) was found in Caprino cheese (ca. 3.6 log CFU g^−1^). Presumptive thermophilic lactobacilli ranged from ca. 4.0 (Pecorino and Cacioricotta) to ca. 5.0 (Caprino and Vaccino cheeses) log CFU g^−1^. Thermophilic streptococci were found at cell densities varying from 3.0 (Caprino) to 4.9 (Pecorino cheese) log CFU g^−1^. The cell density of presumptive enterococci ranged from 2.8 (Pecorino) to 5.2 (Cacioricotta cheese) log CFU g^−1^. The highest (*p* < 0.05) number of presumptive staphylococci was found in Cacioricotta cheese (5.3 log CFU g^−1^), whereas these bacteria were found to have the lowest (*p* < 0.05) number in Caprino and Vaccino cheeses (order of magnitude: 2 log CFU g^−1^). No colonies grown on Baird-Parker could be presumptively attributed to *Staphylococcus aureus*, based on their aspect and the absence of a clear halo surrounding them. *Enterobacteriaceae* were in the order of 2 log CFU g^−1^ in all the cheeses, except for Cacioricotta, where they were found at 3.3 log CFU g^−1^. No presumptive *Pseudomonas* sp. were detected in all the cheeses. Yeasts number was ca. 2.5 log CFU g^−1^ and did not differ (*p* > 0.05) among Caprino, Pecorino and Vaccino cheeses. The lowest (*p* < 0.05) number of yeasts (1.0 log CFU g^−1^) was found in Cacioricotta (Figure 2).

Presumptive mesophilic and thermophilic lactobacilli and cocci were found in NWS cultures used for Caprino (CNWS) and Vaccino (VNWS) cheeses at an order of magnitude of 6.0 log CFU g^−1^, without any statistical (*p* > 0.05) differences between the cultures (data not shown).

### 3.3. Culture-Independent Analysis of the Bacterial Community of TAP Cheeses

**Figure 3:**
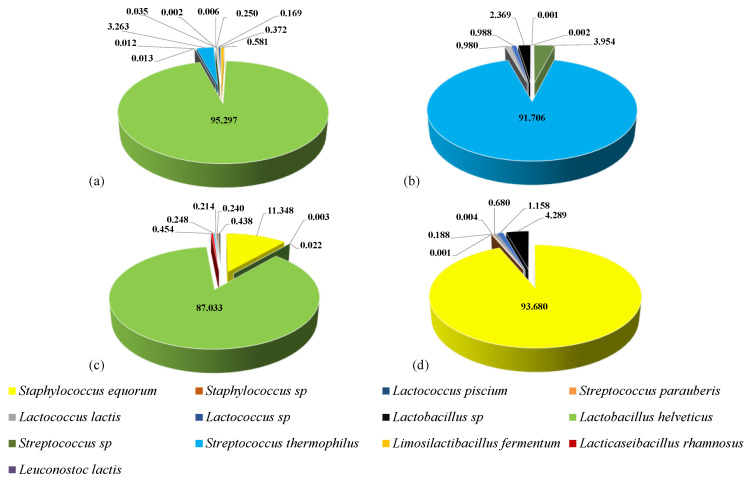
Relative abundance (%) of *Firmicutes* OTU, assigned to the highest possible taxonomic level, found in Caprino (**a**), Pecorino (**b**), Vaccino (**c**), and Cacioricotta (**d**) TAP cheeses.

A total of 740,650 sequences resulted from the 16S metagenetic analysis within the phylum of *Firmicutes*. The number of sequences ranged from ca. 50,000 to ca. 70,000 per sample. The core microbiota of Caprino cheese seemed to be dominated by *Lactobacillus helveticus* with a relative abundance of 95% (Figure 3a).

In this cheese, *Streptococcus thermophilus (Streptococcus salivarius* ssp. *thermophilus)* was also found as the sub-dominant OTU (ca. 3.3% of relative abundance). This species seemed to dominate the bacterial biota of Pecorino cheese, with a relative abundance of ca. 91% (Figure 3b). In this cheese, *Streptococcus* sp. (ca. 3.9%) and *Lactobacillus* sp. (ca. 2.4%) were found as the subdominant OTU. Bacterial biota of Vaccino cheese seemed to be dominated by *L. helveticus* (ca. 87% of relative abundance) and, to a minor extent, *Staphylococcus equorum* (ca. 11.3%) (Figure 3c). In Cacioricotta cheese, *S. equorum* was dominant, with a relative abundance of ca. 94% (Figure 3d). *Lactobacillus* sp. (ca. 4.3%) and *Lactococcus* sp. (ca. 1.2%) were found in this cheese as subdominant OTU. *Lactococcus* sp. and *Lactococcus lactis* were detected in all the cheeses at a very low relative abundance. Other OTU were variously found (at relative abundance below 1%), depending on the cheese: *Limosilactibacillus fermentum* (formerly *Lactobacillus* *fermentum*) and *Lacticaseibacillus rhamnosus* (Caprino and Vaccino cheeses), *Streptococcus parauberis* (Caprino, Pecorino, and Cacioricotta), *Lactococcus piscium* (Caprino and Cacioricotta), *Leuconostoc lactis* (Caprino), and *Staphylococcus* sp. (Vaccino and Cacioricotta cheeses) (Figure 3a–d).

*L. helveticus* dominated the bacterial biota of both the NWS cultures, namely, CNWS (used for Caprino) and VNWS (for Vaccino cheese), where it was detected at a relative abundance of 85.3% and 99.8%, respectively. *L. fermentum* was found as a subdominant OTU (relative abundance of 5.8%) in CNWS (data not shown).

### 3.4. Residual Enzymatic Activities in TAP Cheeses

**Figure 4:**
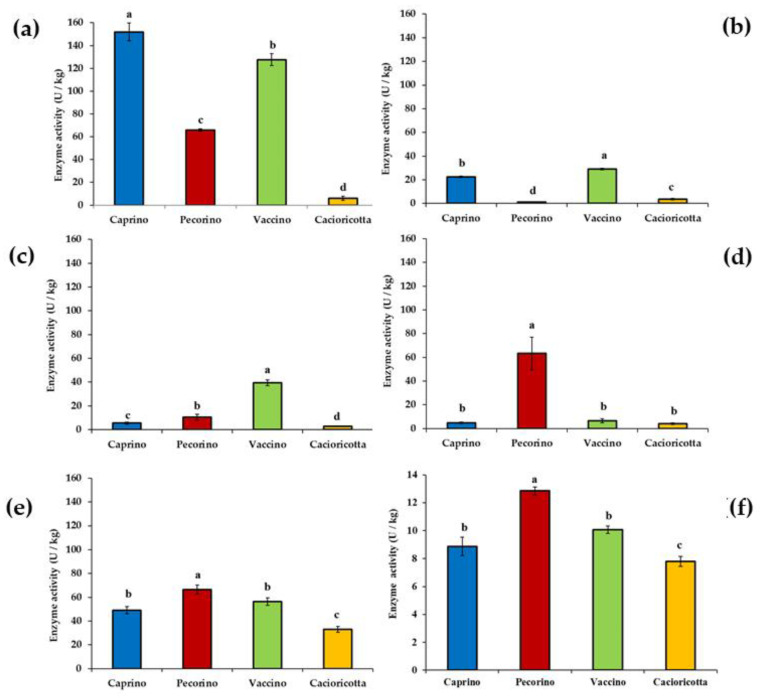
Residual aminopeptidase type N (**a**), iminopeptidase (**b**), endopeptidase type O (**c**), glutamate dehydrogenase (**d**), cystathionine lyase (**e**), and esterase (**f**) activities determined in the water-soluble extracts from Caprino, Pecorino, Vaccino, and Cacioricotta TAP cheeses. For each enzymatic activity, bar-plots marked with different letters (a, b, c, d) are significantly different (*p* < 0.05).

The highest (*p* < 0.05) residual activity for aminopeptidase type N (pepN) was found in Caprino cheese (ca. 150 U kg^−1^, Figure 4a).

Very low pepN activity (ca. 6 U kg^−1^) was found in Cacioricotta cheese. The two other cheeses showed intermediate values. Regarding the iminopeptidase, the highest (*p* < 0.05) activity was found in Vaccino (ca. 29 U kg^−1^), followed by Caprino cheese (Figure 4b). Cacioricotta and Pecorino cheeses showed very low values (1–3.5 U kg^−1^) of iminopeptidase activity. Vaccino cheese was characterized by the highest (*p* < 0.05) activity for type O endopeptidase (ca. 40 U kg^−1^, Figure 4c). Compared to Vaccino, the values of this enzyme activity were from 4-fold (Pecorino cheese) to 10-fold (Cacioricotta cheese) lower. The highest (*p* < 0.05) glutamate dehydrogenase activity was found in Pecorino cheese (ca. 60 U kg^−1^, Figure 4d). The other cheeses showed activity values about 10-fold lower than Pecorino cheese, with no significant (*p* > 0.05) difference among them. Cystathionine lyase ranged from ca. 30 (Cacioricotta) to ca. 65 (Pecorino cheese) U kg^−1^ (Figure 4e). The highest (*p* < 0.05) esterase activity was found in Pecorino (ca. 13 U kg^−1^), followed by Caprino and Vaccino cheeses (without any significant differences between them) (Figure 4f). Cacioricotta cheese showed the lowest (*p* < 0.05) esterase activity (ca. 8 U kg^−1^).

### 3.5. Proteolysis in TAP Cheeses

The urea-PAGE of pH 4.6-insoluble nitrogen fractions showed the presence of protein bands with low electrophoretic mobility in all the cheeses, except for Cacioricotta cheese (Figure S1). These bands presumably corresponded to products of hydrolysis of β-casein (γ-caseins). A large amount of non-hydrolyzed β-casein was found in all the cheeses, except for Pecorino. Protein bands corresponding to α_S1_-casein showed different electrophoretic mobility, depending on the type of milk (goats’, ewes’, cows’ milk) used for cheesemaking. Notwithstanding that all the cheeses, except for Pecorino, showed non-hydrolyzed α_S1_-casein, intense protein bands, corresponding to peptides derived from hydrolysis of α_S1_-casein, were detected, especially in Vaccino cheese (Figure S1).

The RP-FPLC analysis of the pH 4.6-soluble nitrogen fractions highlighted that Pecorino cheese was characterized by the highest number of peptide peaks (ca. 36) and total area (ca. 3600 mAU min) (Figure S2b). About 30 peptide peaks were found for Caprino and Vaccino cheeses, with a total area of ca. 2800 and 2500 mAU min (Figure S2a,c). Cacioricotta cheeses showed ca. 26 peptide peaks and a total area of ca. 1850 mAU min (Figure S2d).

The highest (*p* < 0.05) concentration of total FAA was found in Vaccino cheese (21.45 ± 0.62 mg g^−1^). Compared to Vaccino, Caprino cheese contained a lower (*p* < 0.05) concentration of FAA (16.40 ± 0.541 mg g^−1^, respectively), but higher than Pecorino cheese (6.43 ± 0.52 mg g^−1^). Cacioricotta cheese was characterized by the lowest (*p* < 0.05) level of total FAA (0.09 ± 0.002 mg g^−1^).

### 3.6. VOC Profile of TAP Cheeses

GC-MS was carried out to profile the VOC of the four TAP cheeses object of study. Forty-eight compounds were detected, belonging to seven chemical classes (Table 2).

Eleven carboxylic acids were detected: acetic, butanoic, hexanoic, octanoic, and decanoic acids were the most representative ones. In Pecorino cheese, concentrations of those acids exceeded 100 mg kg^−1^. Among the nine esters found in the cheeses, overall ethyl and butyl esters of octanoic acid were detected at the highest concentrations. Methyl ester of butanoic acid and ethyl ester of hexanoic acid were only detected in Pecorino and Vaccino cheeses. Ethanol (ranging from ca. 3 to ca. 7 mg kg^−1^) and 2,3-butanediol (ca. 2–5 mg kg^−1^) were the most abundant alcohols (12 compounds). Among the six detected ketones, 2-heptanone ranged from ca. 7 (Vaccino) to ca. 28 (Pecorino cheese) mg kg^−1^ and 2-nonanone from ca. 8 (Cacioricotta) to ca. 34 (Pecorino cheese) mg kg^−1^. The most representative aldehyde among the five detected was nonanal, ranging from ca. 1.1 (Caprino and Cacioricotta cheeses) to ca. 1.6 (Pecorino cheese) mg kg^−1^. Three pyrazines were only found in Caprino cheese. The only two lactones detected were caprolactone (only in Pecorino) and dodecalatone (only in Cacioricotta cheese) (Table 2).

Concentrations of VOC in the cheeses were used as entries for PCA. Considering the two first principal components, the various VOC showed different score contributions, depending on the cheese. Pyrazines, butanal-2-methyl and acetaldehyde characterized Caprino cheese (Figure S3). Some ketones (2-heptanone, 2-undecanone, 2-pentanone, and 2-nonanone), alcohols (e.g., 2-propanol and 2-butanol), and, especially, carboxylic acids (e.g., hexanoic and decanoic acids), contributed to the distinct VOC profile of Pecorino cheese (Figure S4). Nonanoic acid, nonanal, 2,3-butanediol, and some esters (ethyl acetate, ethyl ester of hexanoic acid, methyl ester of butanoic acid, and 1-cyclopentylethyl hexanoate) were characteristic VOC of Vaccino cheese (Figure S5). Some alcohols (1-butanol,3-methyl, ethanol, phenylethyl alcohol), ketones (2-butanone and 2-butanone,3-hydroxy, *alias* acetoin), and butanoic acid-3-methyl characterized Cacioricotta cheese (Figure S6).

### 3.7. Correlations between Microbiota and Biochemical Characteristics of TAP Cheeses

**Figure 5:**
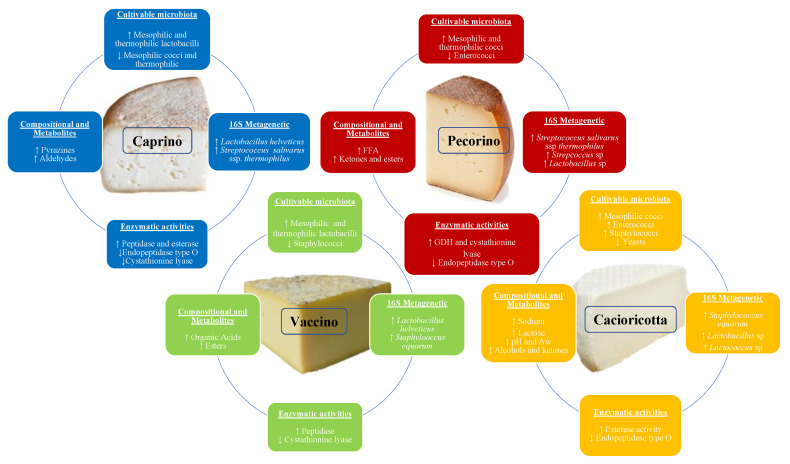
Main distinctive traits (compositional, metabolites, microbiological, enzymatic activities) of Caprino, Pecorino, Vaccino, and Cacioricotta TAP cheeses produced at the same dairy plant. Pointed-up and pointed-down arrows flanking each trait indicate high and low values, respectively. Abbreviations: FFA, free fatty acids; GDH, glutamate dehydrogenase activity.

PCA based on the microbiota (cell densities of bacteria and yeasts, relative abundance of OTU) and biochemical characteristics (pH, Aw, moisture, fat, carbohydrates, enzymatic activity, proteolytic activity, FAA, and VOC) of TAP cheeses accounted for 83% of the total variance (PC1: 49.7% and PC2: 33.3%) (Figure S7). The main distinctive traits of the four cheeses are visually presented in Figure 5. Some high correlations were found among variables. *L. helveticus* was positively correlated with aminopeptidase (r = 0.92), iminopeptidase (0.96), and FAA (0.93). *S. equorum* was positively correlated with butanoic acid,3-methyl (0.92) and acetoin (0.99). Negative correlations were observed between *L. helveticus* and ethanol (r = −0.80), 2-heptanol (−0.79), and 1-hexanol (−0.94), between *S. equorum* and aminopeptidase activity (−0.81), cystathionine lyase activity (−0.86), and most of the VOC. In addition, *S. thermophilus* was negatively (r = −0.62) correlated with iminopeptidase activity.

## 4. Discussion

Four traditional Apulian cheeses (Caprino, Pecorino, Vaccino, Cacioricotta), manufactured at the same dairy plant, were analyzed to highlight their distinctive traits. The influence of house microbiota on cheeses’ traits [36] was minimal because the same equipment and ripening rooms were used for all the cheeses. Yet, the experimental plan suffered from one main limitation, represented by the limited number of samples for each cheese, which hinders gaining a collective overview on each TAP cheese.

The gross composition of the cheeses approached that previously reported for similar types of cheese [32,37,38]. Cacioricotta cheese retained the highest percentage of sodium chloride, in agreement with previous studies [39]. High salt concentration in Cacioricotta could account for its relatively high content in carbohydrates, with special regard to lactose, which was only detected in this cheese. Indeed, the high salt concentration in Cacioricotta may have slowed down lactose catabolism of LAB [40], as reflected by the lowest concentration of lactic acid and highest pH value of the Cacioricotta cheese subjected to study. Although the highest salt concentration was found in Cacioricotta, this cheese was characterized by the highest value of A_w_. This important cheese parameter is affected not only by salt concentration [41] but also by water content and concentration of proteolysis products [42,43,44]. We may hypothesize that the highest value of A_w_ found in Cacioricotta could be due to the shorter ripening time of this cheese (9 months), compared to that applied for the other cheeses (12 months), as well as to low aptitude to syneresis observed for curds originating from milk treated at 90 °C (as was the case for Cacioricotta cheese) [45]. In addition, the low concentration of proteolysis products estimated in this cheese could have determined higher values of A_w_, compared to the other cheeses, showing higher proteolysis levels.

Based on culture-dependent analysis using selective agar media (e.g., MRS, lactose-M17, Slanetz and Bartley), LAB (especially mesophilic) dominated the microbiota of the four cheeses, the object of study. Furthermore, staphylococci represented an additional dominant microbial population in Cacioricotta. *Enterobacteriaceae* and yeasts represented minority microbial populations of cheeses. The use of NWS cultures during the manufacturing of two cheeses (Caprino and Vaccino) only slightly affected the microbial community of those cheeses. This result could be explained considering that most LAB inoculated in cheese milk through the addition of starter cultures declines throughout ripening [46]. Culture-independent analysis showed that *L. helveticus* dominated the bacterial biota of both the NWS (CNWS and VNWS) cultures and the corresponding Caprino and Vaccino cheeses. *L. helveticus* is a thermophilic LAB species, capable of growing at 45 °C, commonly inhabiting NWS cultures, alone or together with *S. thermophilus*, *Lactobacillus delbrueckii,* and *L. fermentum* [6,47,48]. We may hypothesize that *L. helveticus*, originating from NWS cultures, adapted its physiology [49] during the cooking step (45 °C for 10–40 min) of Caprino and Vaccino curds. Then, it survived scalding (a few seconds at 80–85 °C) and probably dominated cheese microbiota during the first days of ripening [46]. It is probable that, although *L. helveticus* could have declined during ripening, it contributed to biochemical pathways characterizing the ripening of Caprino and Vaccino cheeses, thanks to its early cell autolysis, releasing peptidases and other enzymes [50,51,52]. In partial agreement with results from culture-dependent analyses, *S. equorum* seemed to largely dominate the bacterial biota of Cacioricotta cheese object of the current study. This could be explained considering that, after cheese milk had undergone quite a drastic heat treatment that should have inactivated almost all microorganisms inhabiting raw milk, no starter culture was used. In these conditions, the manipulation of cheese by workers (surface dry-salting and daily overturning and washing) could have been one important driver of the microbial community. Given that staphylococci are found on the skin and mucosa of humans [53], the high abundance found for *S. equorum* in the Cacioricotta cheese analyzed in this study could originate from human contamination. *S. equorum* is a common component of the microbial communities of high-salt-fermented foods such as meat products and smear-ripened and semi-hard and hard cheeses [36,54,55]. Although this coagulase-negative staphylococcal species is regarded as a “class of risk 2” pathogen, several strains of *S. equorum* acted as flavor and color enhancers of surface-ripened cheeses [56,57,58]. This species withstands relatively high salt concentrations (such as those encountered in Cacioricotta cheese), uses different carbon sources, and does not harbor gene coding for amino acid decarboxylase, related to release of biogenic amines [59]. Thus far, food-derived strains of *S. equorum* did not show any pathogenic traits [60]. *S. thermophilus* was the dominant bacterial OTU of Pecorino cheese. This result disagreed with the diversity of bacterial community (staphylococci and enterococci, and especially rod- and coccus-shaped LAB) of this cheese emerging from the culture-dependent analysis. In general, discrepancies between the results obtained from the two approaches could derive from some limitations inherent to the DNA-based approach, such as (i) different lysis efficiencies during extraction of DNA, (ii) preferential PCR amplification, and (iii) different numbers of copies of 16S rRNA operons among bacteria [61]. Considering that the Pecorino cheese analyzed in this study was manufactured using pasteurized milk and without starter cultures, we may hypothesize that *S. thermophilus* could have considerably contaminated the equipment of the dairy plant [36,62]. This bacterial species, originating from raw milk and/or starter cultures, could colonize the dairy environment, thanks to its capacity to produce biofilm [63,64]. During cheese ripening, *S. thermophilus* usually decreases over time [54,65,66]. However, it may still inhabit Pecorino cheese, at cell densities of 5–6 log CFU g^−1^, even after 6–12 months of ripening [67,68,69]. Overall, the actual contribution of microbiota to the characteristics of the cheeses object of study remains hazy due to culture-independent analysis performed on DNA (instead of RNA) and only targeted at the *Firmicutes* bacterial phylum.

In this study, the highest peptidase activities were found in either Caprino or Vaccino cheeses, probably due to NWS cultures, which add microbial biomass and related enzyme activities to cheese [70]. In line with these findings, *L. helveticus* as the dominant LAB species detected in Caprino and Vaccino cheeses can count on a potent proteolytic system producing short peptides and liberating FAA from the casein matrix [71]. Indeed, positive correlations were found between *L. helveticus* and some variables related to the proteolysis level of cheeses. The lowest enzyme activities, found in Cacioricotta cheese, could be caused by the high salt concentration of this cheese. Indeed, an increase in salt concentration lowered some peptidase activities [72]. Contrarily, Møller et al. [73] reported higher levels of aminopeptidase activities in Cheddar cheese with increasing salt concentrations. The lowest iminopeptidase activity (pro-pNA) detected in Pecorino could be ascribed to the dominance of *S. thermophilus*, as remarked by the negative correlation between this species and enzyme activity. *S. thermophilus* does not harbor the proline iminopeptidase within its enzymatic portfolio [70,74]. Enzymes involved in the catabolism of FAA and esterase showed the highest activity in Pecorino cheese. In particular, the highest GDH activity could be ascribed to *S. thermophilus*, as evidenced by a positive correlation between these variables, and in agreement with Peralta et al. [75] Caprino and Vaccino cheeses were characterized by a relatively high concentration of FAA, consistently with their high peptidase activities. The low concentration of FAA and total area and number of pH 4.6-soluble peptides found in Cacioricotta cheese reflected its low peptidase activities, as well as the minor hydrolysis degree of β-casein estimated through urea-PAGE. Salt inhibits hydrolysis of β-casein [73]. Vaccino cheese showed hydrolysis products from α_S1_-casein, probably because the proteolytic enzymes of microbial rennet could have a higher affinity towards cow α_S1_-casein, compared to the same fraction of other milk species [76].

Besides microbiological, enzymatic and proteolytic traits, we compared the VOC profiles of the four TAP cheeses, using the solid phase micro-extraction followed by GC-MS, well known for two decades as a reliable tool to detect some flavor compounds that, although at low concentrations, strongly impact on aroma and odor of cheese [77]. VOC profiling highlighted that carboxylic acids (free fatty acids, FFA), resulting from hydrolysis of triacylglycerols, were the VOC found at the highest concentrations. Although they (especially those with less than 12 carbon atoms) affect the odor and taste of cheese, ketones, alcohols, and esters, produced upon catabolism of FFA, have an even greater influence on sensory traits, due to their lower flavor threshold [78]. Methyl ketones (alkan-2-ones), the VOC class found at the highest (after FFA) concentration in our cheeses, derive from β-oxidation and decarboxylation of carboxylic acids [78]. For instance, 2-heptanone and 2-nonanone derive from octanoic and decanoic acids, respectively. They confer fruity, floral, musty, or “blue cheese” notes [79]. Among alcohols, our cheeses were rich in ethanol (from lactose fermentation and/or amino acid metabolism) [80,81] and some secondary alcohols. The latter may result from the reduction of methyl ketones [82]. For instance, 2-pentanol could derive from 2-pentanone. Other secondary alcohols, such as 1-butanol,3-methyl, and phenylethyl alcohol (*alias* 2-phenylethanol), could derive from the microbial driven Ehrlich pathway of FAA [83]. Esters may derive from either esterification of FFA with ethanol or the transfer of fatty acid from glycerides (especially mono- and di-glycerides) to ethanol (alcoholysis) [84]. They have a 10-fold lower flavor threshold than their alcohol precursor, often ethanol [85], and confer sweet, fruity, and floral notes to cheese [86].

All the cheeses subjected to this study showed distinct VOC profiles. This depends on milk type and quality, microbial activity [87,88] and processing operations. Specific dairy processing affects the VOC profile of a cheese so much that this distinctive trait can be used as marker of product and process [89]. In a recent study, several VOC characterizing the profile of model cheeses were associated, through linear discriminant analysis, to cheese milk obtained from different breeding systems [90]. In the present study, Pecorino was the richest cheese in VOC related to lipolysis, i.e., FFA, ketones, and esters. Given that this cheese was produced from pasteurized milk and without starters, we might exclude milk lipoprotein lipase and enzymes from microbial starters acting as lipolytic agents of Pecorino cheese. A possible explanation worthy of investigation could be the esterase activity of *S. thermophilus*, which seemed to dominate the bacterial biota of Pecorino cheese. Among the strains of dairy origin and belonging to seven different LAB species, those of *S. thermophilus* showed the highest esterase activity in alcoholysis [84]. Given that esterase from *S. thermophilus* showed ester-synthesizing capacity [84], we may hypothesize that *S. thermophilus* could have been the source of esterase contributing to lipolysis and catabolism of FFA in the Pecorino cheese object of study. The VOC profile of Caprino (from goats’ milk) included pyrazine derivatives as a distinctive trait. Only a few studies have assessed the presence of pyrazines in goats’ milk cheeses [77,91]. Although pyrazines have been frequently detected in ewes’ milk cheeses [38,92,93], in our study this class of compounds was not detected in Pecorino cheese (from ewes’ milk). Pyrazines confer to cheese a mild to intense roast nut aroma, and originate during Maillard reaction, which depends on many factors, such as temperature and the availability of sugars [77]. Caprino, along with Vaccino cheese as the object of the current study, were characterized by high levels of FAA and peptidase activities, which may have impacted on pyrazines formation in Caprino cheese, in agreement with a previous study [94]. Although we found that Caprino and Vaccino cheeses shared several traits (e.g., high abundance of *L. helveticus* and level of peptidase activity), the VOC profiles markedly differed between these two cheeses, Caprino cheese being richer in, besides pyrazines, aldehydes, some medium chain fatty acids and their derived ethyl esters. We may hypothesize that this could be due to differences in the type of milk and in the cheese-making protocols. 1-Butanol,3-methyl and phenylethyl alcohol were the distinctive VOC of Cacioricotta cheese. In addition, this cheese was characterized also by the highest concentration of 3-hydroxy-2-butanone (acetoin), originating from either diacetyl (2,3-butanedione) reduction or metabolism of pyruvate, lactose, or citrate [95]. 3-methyl butanoic acid was another distinctive compound of Cacioricotta cheese. The highest amount of acetoin and 3-methyl butanoic acid found in Cacioricotta could be ascribed to the presence of *S. equorum*, which has been recognized as a potential producer of acetoin and branched chain fatty acids, which are important flavor compounds [59,96].

## 5. Conclusions

The results of this study highlight that, even if manufactured at the same dairy plant, the four TAP cheeses clearly distinguished one from each other, especially due to the presence/absence of the thermal treatment of milk, use of NWS cultures, manipulation during ripening and, supposedly, milk species. In addition, some VOC could be distinctive traits of cheeses. Future studies aiming to valorize and safeguard TAP cheeses will have to consider cheeses of the same variety sampled at different dairy plants throughout manufacturing and ripening processes.

## Figures and Tables

**Table 1:** Gross chemical composition (expressed in percentage, *w/w*), values of water activity (A_w_), and pH of Caprino, Pecorino, Vaccino, and Cacioricotta TAP cheeses.

	Caprino	Pecorino	Vaccino	Cacioricotta
Moisture (%)	34.18 ^b^ ± 1.18	30.98 ^cd^ ± 1.59	31.99 ^c^ ± 1.32	38.10 ^a^ ± 1.24
Fat (%)	29.42 ^b^ ± 0.50	35.14 ^a^ ± 0.5	34.25 ^ab^ ± 0.5	27.08 ^c^ ± 0.5
Protein (%)	26.14 ^ab^ ± 0.30	27.09 ^a^ ± 0.20	24.54 ^b^ ± 0.30	22.13 ^c^ ± 0.30
Carbohydrates (%)	0.71 ^c^ ± 0.20	1.40 ^b^ ± 0.20	1.43 ^b^ ± 0.30	4.20 ^a^ ± 0.20
NaCl (%)	0.61 ^c^ ± 0.20	0.91 ^b^ ± 0.20	0.90 ^b^ ± 0.20	6.23 ^a^ ± 0.20
A_w_	0.879 ^b^ ± 0.05	0.868 ^c^ ± 0.03	0.858 ^d^ ± 0.04	0.905 ^a^ ± 0.07
pH	5.22 ^b^ ± 0.02	5.23 ^b^ ± 0.04	5.30 ^b^ ± 0.03	5.93 ^a^ ± 0.03

^a–d^ Values in the same row with different letters are significantly different (*p* < 0.05).

**Table 2:** Concentration (in mg kg^−1^) of volatile organic compounds detected in Caprino, Pecorino, Vaccino, and Cacioricotta TAP cheeses.

Compounds	Caprino	Pecorino	Vaccino	Cacioricotta
Acetic acid	36.170 ^b^	84.573 ^a^	53.141 ^b^	4.558 ^c^
Butanoic acid	113.145 ^b^	266.107 ^a^	87.824 ^b^	22.280 ^c^
Butanoic acid, 3-methyl-	0.585 ^b^	0.402 ^c^	0.447 ^bc^	0.915 ^a^
Pentanoic acid	1.404 ^b^	3.170 ^a^	0.982 ^c^	0.649 ^d^
Hexanoic acid	221.612 ^b^	485.377 ^a^	95.968 ^c^	49.290 ^d^
Heptanoic acid	3.548 ^b^	15.290 ^a^	0.378 ^c^	0.502 ^c^
Octanoic acid	153.853 ^b^	344.211 ^a^	13.183 ^d^	20.891 ^c^
Nonanoic acid	6.257 ^c^	7.722 ^a^	7.891 ^a^	3.638 ^d^
Decanoic acid	58.208 ^b^	136.895 ^a^	3.186 ^c^	5.905 ^c^
Decenoic acid	1.197 ^b^	5.427 ^a^	0.212 ^c^	0.132 ^c^
Dodecanoic acid	2.368 ^b^	6.826 ^a^	0.440 ^c^	0.306 ^c^
Total acids	598.35	1356	263.65	109.06
Acetic acid, ethyl ester	0.232 ^b^	0.425 ^a^	0.314 ^ab^	0.256 ^b^
Butanoic acid, methyl ester	n.d. ^1^	0.612 ^b^	0.981 ^a^	n.d.
Butanoic acid, ethyl ester	1.390 ^b^	3.205 ^a^	1.101 ^bc^	0.786 ^c^
Hexanoic acid, ethyl ester	n.d.	1.155 ^b^	2.183 ^a^	n.d.
Octanoic acid, ethyl ester	3.014 ^b^	7.249 ^a^	1.666 ^c^	1.380 ^c^
Decanoic acid, ethyl ester	1.042 ^b^	4.076 ^a^	0.229 ^c^	0.226 ^c^
Butanoic acid, butyl ester	0.771 ^b^	2.910 ^a^	0.753 ^b^	0.321 ^b^
Octanoic acid, butyl ester	4.460 ^b^	6.420 ^a^	1.169 ^c^	0.883 ^c^
Hexanoic acid, 1-cyclopentylethyl ester	0.396 ^b^	0.406 ^c^	0.535 ^a^	0.234 ^d^
Total esters	11.3	26.46	8.93	4.08
2-Propanol	n.d.	0.931 ^a^	n.d.	n.d.
Ethanol	2.506 ^c^	4.215 ^b^	3.167 ^bc^	7.204 ^a^
2-Butanol	n.d.	2.042	n.d.	n.d.
2-Pentanol	0.499 ^c^	6.616 ^a^	0.614 ^b^	n.d.
1-Butanol, 3-methyl-	0.673 ^c^	n.d.	1.097 ^b^	5.794 ^a^
2-Heptanol	0.392 ^c^	1.410 ^a^	0.518 ^c^	0.737 ^b^
1-Hexanol	n.d.	0.473 ^a^	n.d.	0.291 ^b^
2-Ethylhexanol	0.574 ^a^	0.292 ^c^	0.323 ^c^	0.405 ^b^
2-Nonanol	0.389 ^c^	0.736 ^a^	0.514 ^b^	0.442 ^c^
2,3-Butanediol	3.040 ^b^	2.463 ^b^	5.146 ^a^	2.138 ^b^
Phenylethyl alcohol	n.d.	n.d.	n.d.	0.392 ^a^
2-Furanmethanol	0.382 ^a^	n.d.	n.d.	n.d.
Total alcohols	8.46	19.18	11.38	15.27
2-Butanone	n.d.	0.449 ^b^	0.427 ^b^	0.601 ^a^
2-Pentanone	5.769 ^b^	10.458 ^a^	2.195 ^c^	1.062 ^d^
2-Heptanone	15.373 ^b^	28.399 ^a^	6.658 ^c^	7.901 ^c^
2-Butanone, 3-hydroxy- (Acetoin)	1.679 ^b^	1.605 ^b^	1.630 ^b^	5.273 ^a^
2-Nonanone	19.974 ^b^	34.366 ^a^	19.541 ^b^	7.763 ^c^
2-Undecanone	2.212 ^b^	7.200 ^a^	2.930 ^b^	0.185 ^c^
Total ketones	45.01	82.48	33.38	22.78
Acetaldehyde	1.081 ^a^	n.d.	0.298 ^b^	n.d.
Butanal, 2-methyl-	0.237 ^a^	n.d.	n.d.	n.d.
Butanal, 3-methyl-	0.755 ^a^	0.344 ^d^	0.598 ^b^	0.390 ^c^
Nonanal	1.116 ^b^	1.633 ^a^	1.469 ^ab^	1.171 ^b^
Benzaldehyde	0.938 ^a^	0.831 ^b^	n.d.	0.249 ^c^
Total aldehydes	4.13	2.81	2.37	1.81
Pyrazine, 2,5-dimethyl-	0.480 ^a^	n.d.	n.d.	n.d.
Pyrazine, 2,6-dimethyl-	1.226 ^a^	n.d.	n.d.	n.d.
Pyrazine, trimethyl-	0.438 ^a^	n.d.	n.d.	n.d.
Total pyrazines	2.14	n.d.	n.d.	n.d.
Caprolactone	n.d.	2.669 ^a^	n.d.	n.d.
Dodecalactone	n.d.	n.d.	n.d.	0.235 ^a^
Total lactones	n.d.	2.67	n.d.	0.23

^1^ n.d., not detected. ^a–d^ Values in the same row with different letters are significantly different (*p* < 0.05).

## Data Availability

Data are contained within this article and related Supplementary Materials. The data presented in this study are openly available in BioProject PRJNA776555. https://dataview.ncbi.nlm.nih.gov/object/PRJNA776555?reviewer=10sq6geavcbqt987l9q8rtr1jv.

## Supplementary Materials

The following supporting information can be downloaded at: https://www.mdpi.com/2304-8158/11/3/425/s1, Figure S1: Urea-Polyacrylamide Gel Electrophoresis (PAGE) of pH 4.6-insoluble nitrogen fractions extracted from Caprino, Pecorino, Vaccino, and Cacioricotta TAP cheeses. Lanes: 1, sodium caseinate from goats’ milk; 2, Caprino cheese; 3, sodium caseinate from ewes’ milk; 4, Pecorino cheese; 5, sodium caseinate from cows’ milk; 6, Vaccino cheese; 7, Cacioricotta cheese. Figure S2: Reverse-Phase Fast Protein Liquid Chromatography (RP-FPLC) peptides profiles of the pH 4.6-soluble nitrogen fractions of Caprino (a), Pecorino (b), Vaccino (c), and Cacioricotta (d) TAP cheeses. Figure S3: Loadings of the first two principal components after the principal component analysis (PCA) of the volatile organic compounds detected in Caprino TAP cheese. Figure S4: Loadings of the first two principal components after the principal component analysis (PCA) of the volatile organic compounds detected in Pecorino TAP cheese. Figure S5: Loadings of the first two principal components after the principal component analysis (PCA) of the volatile organic compounds detected in Vaccino TAP cheese. Figure S6: Loadings of the first two principal components after the principal component analysis (PCA) of the volatile organic compounds detected in Cacioricotta TAP cheese. Figure S7: Score and loading plots of first and second principal components after principal component analysis on cell densities of bacteria and yeasts, the relative abundance of OTU and biochemical characteristics (pH, Aw, moisture, fat, carbohydrates, enzymatic activities, total area of peptide peaks, FAA, and VOC) of Caprino, Pecorino, Vaccino, and Cacioricotta TAP cheeses.
