# Supplementary figures and images for: Distinctive Traits of Four Apulian Traditional Agri-Food Product (TAP) Cheeses Manufactured at the Same Dairy Plant
Source: Foods. 2022 Feb 1;11(3):425. doi: 10.3390/foods11030425 (PMC8834160; doi:10.3390/foods11030425)

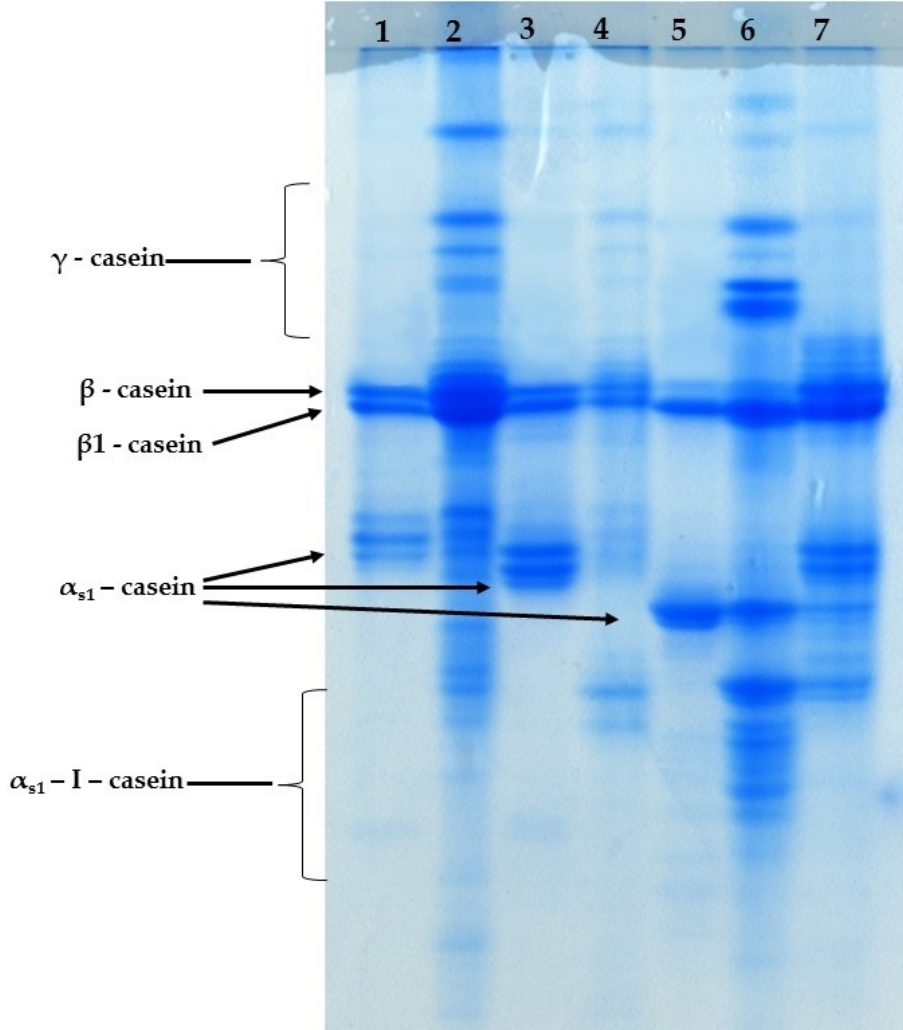

Supplement: Supplementary file 1 [file foods-11-00425-s001.zip › foods-1470862-SI/Supplementary Figures Celano et al.,2021/figure S1.pdf]

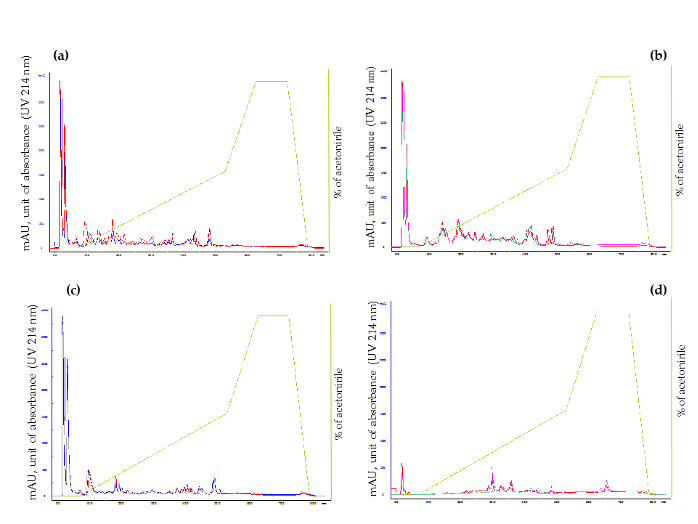

Supplement: Supplementary file 1 [file foods-11-00425-s001.zip › foods-1470862-SI/Supplementary Figures Celano et al.,2021/figure S2.png]

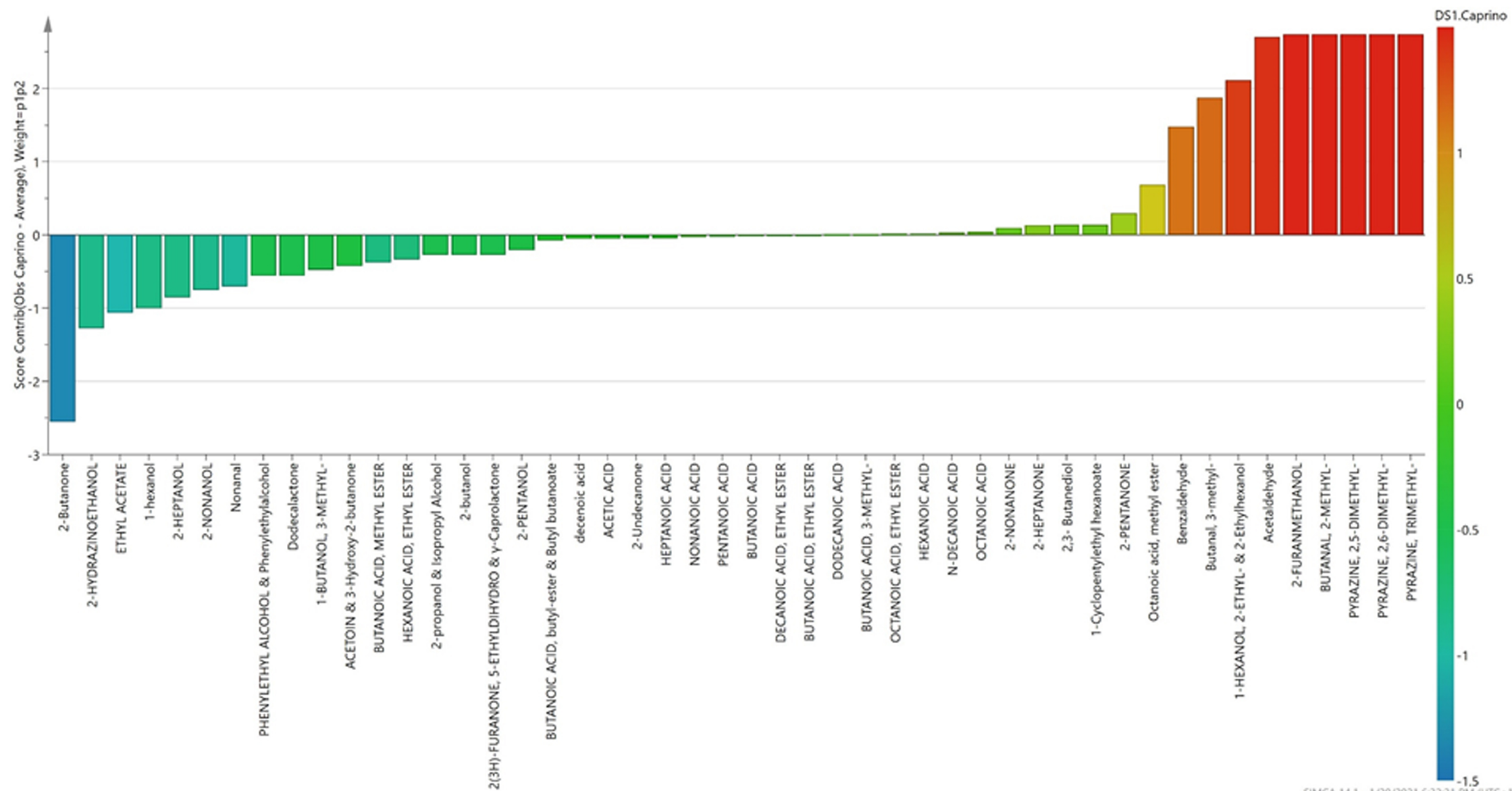

Supplement: Supplementary file 1 [file foods-11-00425-s001.zip › foods-1470862-SI/Supplementary Figures Celano et al.,2021/figure S3.pdf]

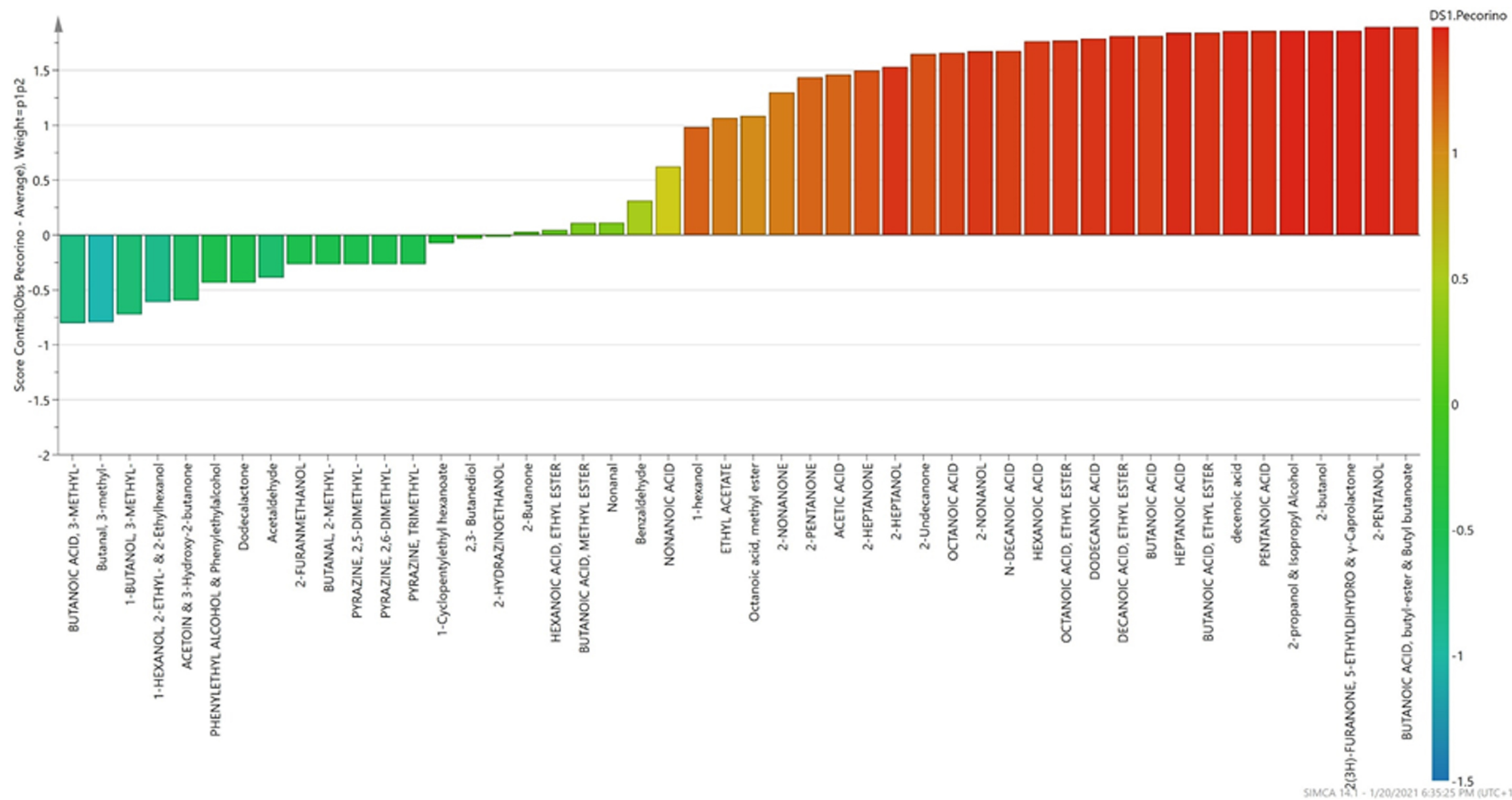

Supplement: Supplementary file 1 [file foods-11-00425-s001.zip › foods-1470862-SI/Supplementary Figures Celano et al.,2021/figure S4.pdf]

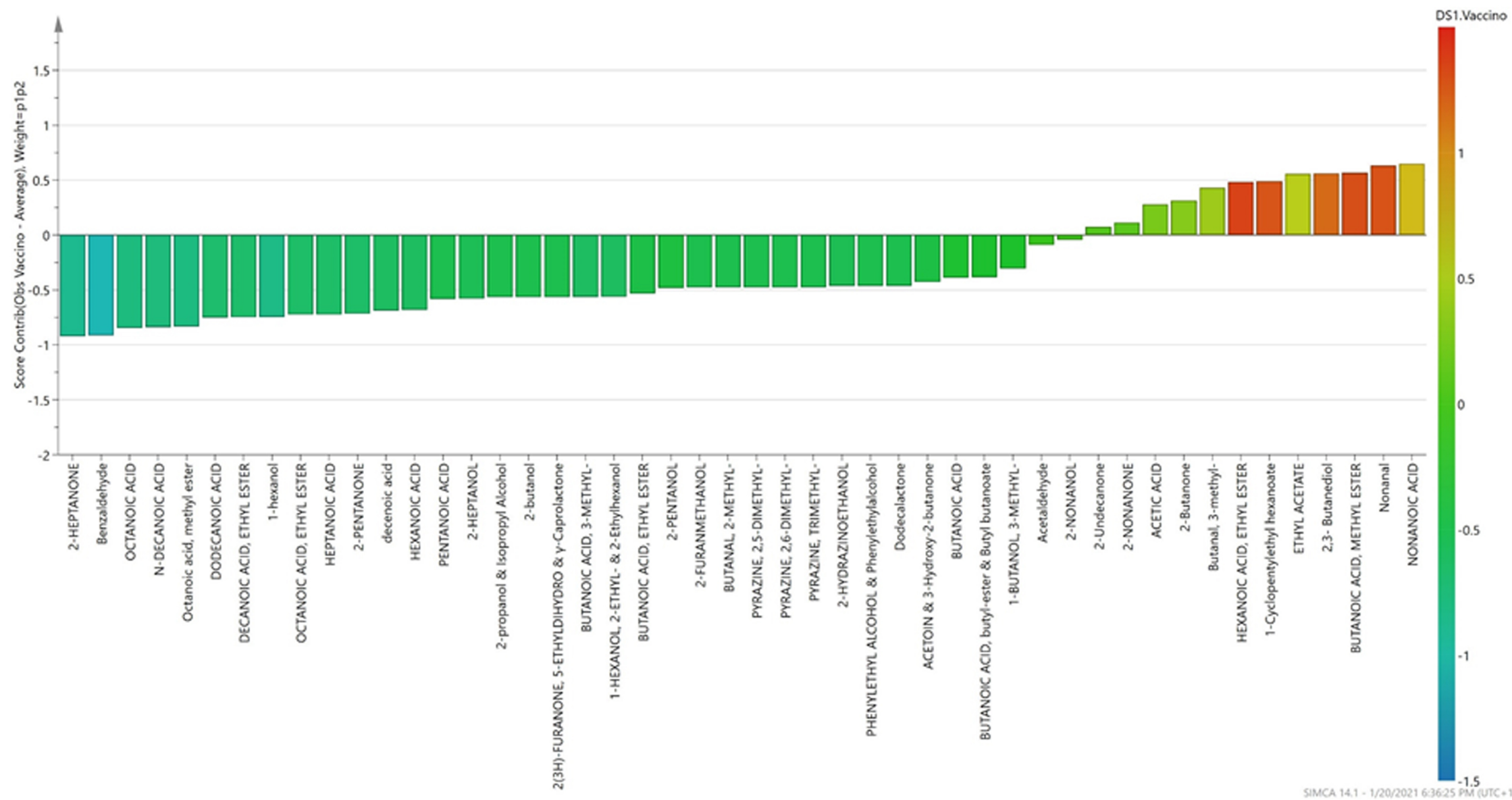

Supplement: Supplementary file 1 [file foods-11-00425-s001.zip › foods-1470862-SI/Supplementary Figures Celano et al.,2021/figure S5.pdf]

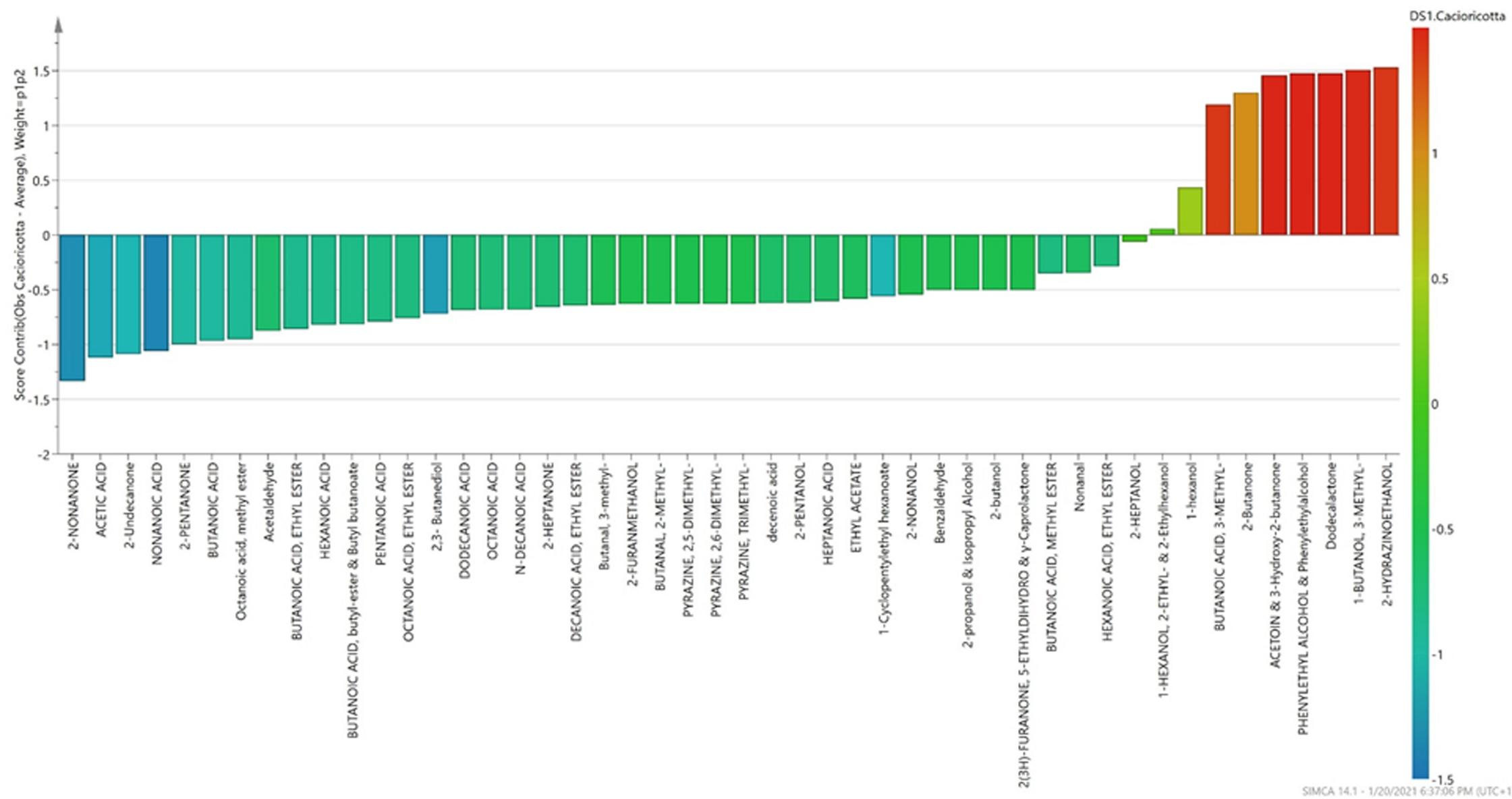

Supplement: Supplementary file 1 [file foods-11-00425-s001.zip › foods-1470862-SI/Supplementary Figures Celano et al.,2021/figure S6.pdf]

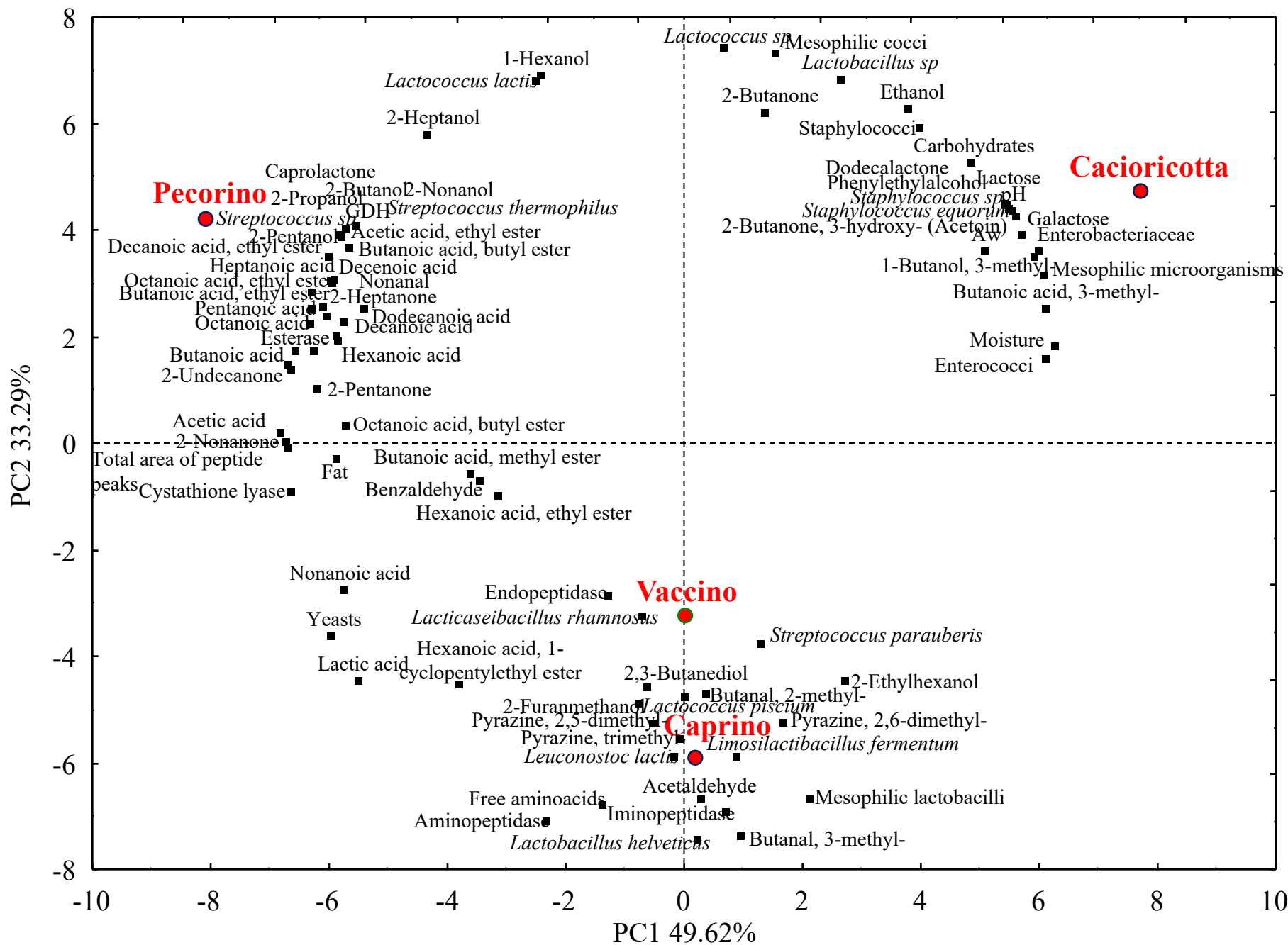

Supplement: Supplementary file 1 [file foods-11-00425-s001.zip › foods-1470862-SI/Supplementary Figures Celano et al.,2021/figure S7.pdf]
